# 

**Published:** 1896-02

**Authors:** 


### TABLE OF CONTENTS.

ORIGINAL COMMUNICATIONS. PAGE

Electrical Osmosis for the Treatment of Living Dentine. By HENRY W. GILLETT, D.M.D., Newport, R. I. . . . . . . . . . . . . . . . . . . . 65

Hypnotism: Its Value to the Dental Specialist. By DR. GEORGE F. GRANT, Boston, Mass. . . . . . . . . . . . . . . . . . . . . . . . . 83

Dental Examining Boards in the United States and Preliminary Education. By G. CARLETON BROWN, D.D.S., Elizabeth, N. J. . . . . . . . . 87

"Resistibility." By DR. G. A. MILLS, New York . . . . . . . . . . . 94

REPORTS OF SOCIETY MEETINGS.

American Academy of Dental Science . . . . . . . . . . . . . . . . . . 95

The New York Institute of Stomatology . . . . . . . . . . . . . . . . 105

EDITORIAL.

The War on the Dental Colleges . . . . . . . . . . . . . . . . . . . . 122

Explanation . . . . . . . . . . . . . . . . . . . . . . . . . . . . . . 126

BIBLIOGRAPHY . . . . . . . . . . . . . . . . . . . . . . . . . . . . . 126

OBITUARY.

Thomas H. Chandler—Resolutions of Respect . . . . . . . . . . . . . . 128

John De Haven White, M.D., D.D.S. . . . . . . . . . . . . . . . . . . 129

In Memory of Professor James E. Garretson . . . . . . . . . . . . . . 131

Elisha G. Tucker, M.D. . . . . . . . . . . . . . . . . . . . . . . . . 131

CURRENT NEWS.

Odontographic Society of Chicago . . . . . . . . . . . . . . . . . . . 132

Topics for Discussion by State and Local Organizations . . . . . . . . 132

